# The Effect of Polysialic Acid Expression on Glioma Cell Nano-mechanics

**DOI:** 10.1007/s12668-016-0192-2

**Published:** 2016-01-25

**Authors:** Colin A. Grant, Peter C. Twigg, Rida F. Saeed, Gary Lawson, Robert A. Falconer, Steven D. Shnyder

**Affiliations:** Advanced Materials Engineering, Faculty of Engineering and Informatics, University of Bradford, Bradford, BD7 1DP UK; Institute of Cancer Therapeutics, Faculty of Life Sciences, University of Bradford, Bradford, BD7 1DP UK

**Keywords:** AFM, Nano-mechanics, Cell elasticity, Tumour dissemination, Polysialic acid

## Abstract

Polysialic acid (polySia) is an important carbohydrate bio-polymer that is commonly over-expressed on tumours of neuroendocrine origin and plays a key role in tumour progression. polySia exclusively decorates the neural cell adhesion molecule (NCAM) on tumour cell membranes, modulating cell-cell interactions, motility and invasion. In this preliminary study, we examine the nano-mechanical properties of isogenic C6 rat glioma cells—transfected cells engineered to express the enzyme polysialyltransferase ST8SiaII, which synthesises polySia (C6-STX cells) and wild-type cells (C6-WT). We demonstrate that polySia expression leads to reduced elastic and adhesive properties but also more viscoelastic compared to non-expressing wild-type cells. Whilst differences in cell elasticity between healthy and cancer cells are regularly assigned to changes in the cytoskeleton, we show that in this model system, the change in properties at the nano-level is due to the polySia on the transfected cell membrane surface.

Polysialic acid (polySia) is a carbohydrate homopolymer of α-2,8-linked sialic acid residues that is a key component of the tumour glycocalyx [1]. polySia is exclusively expressed on the neural cell adhesion molecule (NCAM) and plays a key role in controlling tumour cell growth and differentiation by modulating NCAM signalling at cell-cell contacts, resulting in altered cell adhesion and an increase in cell motility and invasion [2]. polySia plays an important role in neural development and plasticity [3], but crucially is re-expressed on tumours of neuroendocrine origin, including glioma, neuroblastoma, lung cancer and multiple myeloma [2]. It was recently demonstrated in a pair of isogenic C6 rat glioma cell lines that cells transfected with the enzyme responsible for synthesis of polySia, (polysialyltransferase ST8SiaII or ‘STX’) (Fig. 1), migrate at a higher rate in an in vitro scratch assay than wild-type cells [4]. In addition, these cells demonstrate increased invasion across the *corpus callosum* of the brain in vivo [5]. Whilst there is good understanding of the role played by polySia in cell adhesion, migration and invasion, the effect of polySia expression on cell elasticity has not been explored. In this study, we investigate this using the C6 isogenic cell line pair. Changes in cell elasticity may provide new mechanisms for diagnostic and therapeutic clinical intervention [6].

**Fig. 1:**
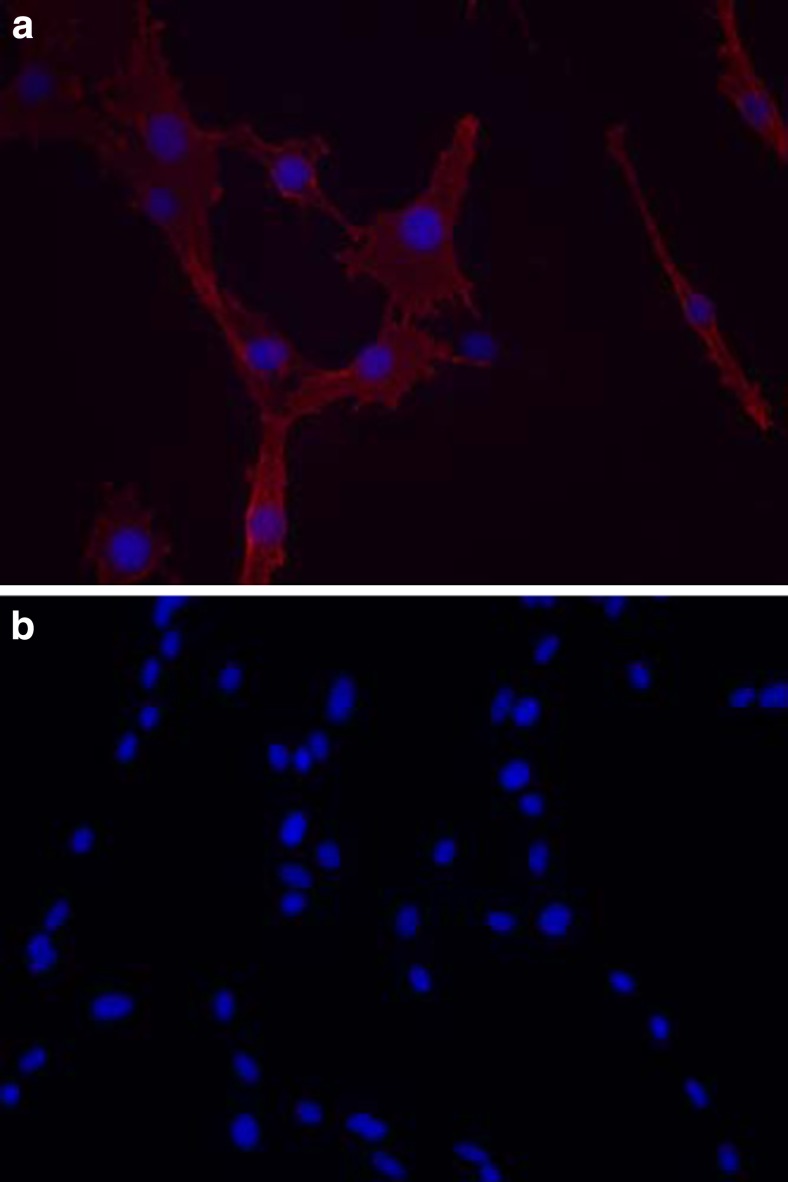
Immunofluorescence images of isogenic C6 rat glioma lines immunolabelled with anti-polySia antibody mAb 735. Cells transfected with ST8SiaII (**a**) clearly demonstrate polySia expression at the cell membrane, whereas no expression is seen for C6-WT cells (**b**)

Atomic force microscopy (AFM) has become a very important technique for examining soft, biological matter [7]. As the AFM probes are parabolic, they can be used as a precision nano-indenter to ascertain the nano-mechanical properties of a region of interest [8–10]. AFM has been regularly used to examine the nano-mechanical properties of a wide range of cells, including fibroblasts, cancer cells and bacteria [6, 11–14]. Changes in the mechanical behaviour of cells have long been associated with a variety of pathologies [15].

The methodology to transfect glioma cells with ST8SiaII is reported elsewhere [5]. The C6-STX and C6-WT glioma cell lines were maintained in alpha-MEM medium supplemented with 10 % foetal bovine serum. For AFM live cell imaging, 4 × 10^3^ cells were seeded onto 22 mm × 22 mm glass coverslips in six-well plates and allowed to adhere overnight before testing. For immunocytochemistry, cells were fixed with pre-cooled methanol at −20 °C prior to immunolabelling with mouse monoclonal antibodies to either polySia or β-actin, with detection using a TRITC-labelled rabbit anti-mouse secondary and a DAPI nuclear counterstain.

AFM force mapping techniques were carried out using an Asylum Research MFP-3D (Santa Barbara, USA) with a commercially available BioHeater stage to maintain the aqueous scanning media at 37 °C. Cantilevers were aligned over a cell using the AFM optics (×10). After scanning a cell, a force map array of 16 × 16 indentations, at loads 1 nN with a tip velocity of 1 μm/s, was made. This was repeated on 10 cells for each cell type (*n* = 2560 force plots per cell type). Force (*F*) vs indentation (*h*) plots were subsequently fitted with a simple Hertzian conical relationship (Eq. 1) to extract the elastic modulus (*E*), using a half cone angle of *α* = 36° and Poisson ratio (*ν*) of 0.5.

1 $$ F=\frac{2}{\pi}\left[\frac{E}{1-{v}^2}\right]{h}^2 \tan \alpha $$

Figure 2a shows a schematic of a force-indentation profile along with other regions of interest that have important mechanical data of adhesion and hysteresis. The AFM cantilever is aligned to a cell under aqueous conditions and at 37 °C, using the AFM optics (inset Fig. 2a). Representative force curves made on each cell type is shown in Fig. 2b, where the stiffer WT has a greater gradient than the STX cell. An interesting feature occurred a few times on some STX cells, which exhibited a jump-in feature (inset Fig. 2b). This could be either a jump to contact, which is not often seen under aqueous conditions, or a possible penetration event of the polySia on the cell membrane (approx. 100 nm). Adhesion describes the amount of force required to separate the AFM tip and cell surface, whilst the hysteresis (the area bound between the loading and unloading curves) demonstrates the amount of energy lost to the cell from indentation. Hysteresis is directly related to the viscoelastic properties. The distribution of modulus and adhesion results from each of the two cell types is shown in Figs. 2c–d.

**Fig. 2:**
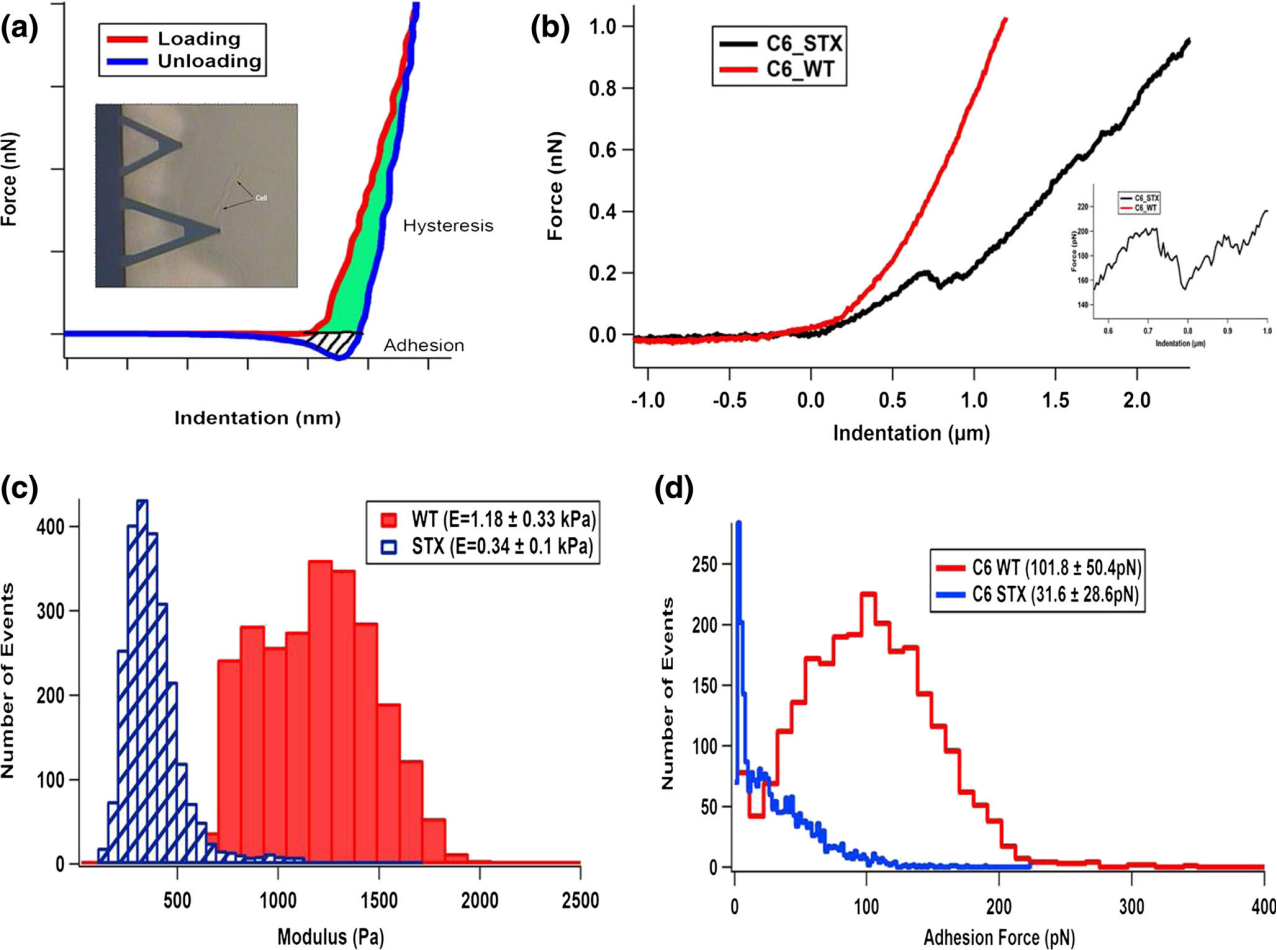
**a** Schematic diagram of a force curve showing key parameters; (*inset*) AFM optics for locating appropriate cells. **b** Typical force curves on WT and STX; (*inset*) zoom in on the “jump-in” on the STX force curve. **c** Moduli distributions. **d** Adhesion force distribution

The mean modulus for the wild-type glioma cells (C6-WT) was 1.2 ± 0.33 kPa, whilst for the polySia-expressing C6-STX cells, the modulus was a factor of 3 lower at 0.34 ± 0.1 kPa. From the same data, the AFM software is able to extract the degree of adhesion between the silicon nitride probe and each cell surface. The C6-WT cells had an adhesion force mean of 101.8 ± 50.4 pN, and the C6-STX cells, as expected, had a reduced adhesion force of 31.6 ± 28.6 pN. The hysteresis or work of indentation is the area between the loading and unloading curves and above the zero force. Here, the C6-WT cells had a mean of 681 ± 145.4 aJ and the C6-STX cells had a mean of 729 ± 117.3 aJ. Overall, these data show that the polySia-expressing C6-STX cells are more compliant, have reduced adhesion, and are more dissipative than the wild-type cells.

To the best of our knowledge, there is no other published nano-mechanical analysis of C6 glioma cells or indeed polySia-expressing cells, making comparisons of our results difficult. However, there is a range of papers describing AFM-based nano-indentation that suggest that the modulus for cells is in the region of 1–5 kPa [6, 12, 16–18]. Furthermore, mechanical studies that use a microfluidic approach on a variety of similar-sized cell types demonstrated a range of moduli from 210 Pa to 23 kPa [19]. Recently, it was shown that the nano-mechanical properties of cell nuclei can be determined using a modified AFM probe. Here, these authors showed that the modulus of nucleus from highly metastatic bladder cancer cell line T24 is lower than its less metastatic counterpart RT4 [20]. Their results also showed that the nucleus does have a higher stiffness than its surrounding cytoplasm/cell membrane. In our study, it is likely that the cell nucleus of the isogenic cell types is mechanically identical, suggesting that the differences we found in the nano-mechanics are likely to originate from the cell-surface polySia expression. Whilst we did not directly measure nuclear nano-mechanics, the region of indentation was over the nucleus, which means its stiffness will be accounted for in our force measurements.

For the use of Hertzian-based indentations, the general assumption is that the material being indented is isotropic and homogeneous. This is a poor description of complex biological systems such as cells [21]. However, as our indentation analysis is directly comparing cells under the same conditions, using the same loading parameters, at similar locations, we believe that the reported nano-mechanical differences between the isogenic cells are genuine.

A number of publications show that cancer cells exhibit a reduced elastic modulus as compared with non-malignant cells [6, 22–24]. Alterations and changes to actin filaments are suggested to be the main reasons behind this apparent softening in cellular modulus [25]. In this study, no discernible differences were observed for immunolabelling patterns for β-actin when viewed under a laser confocal microscope (Zeiss LSM 710) (Fig. 3).

**Fig. 3:**
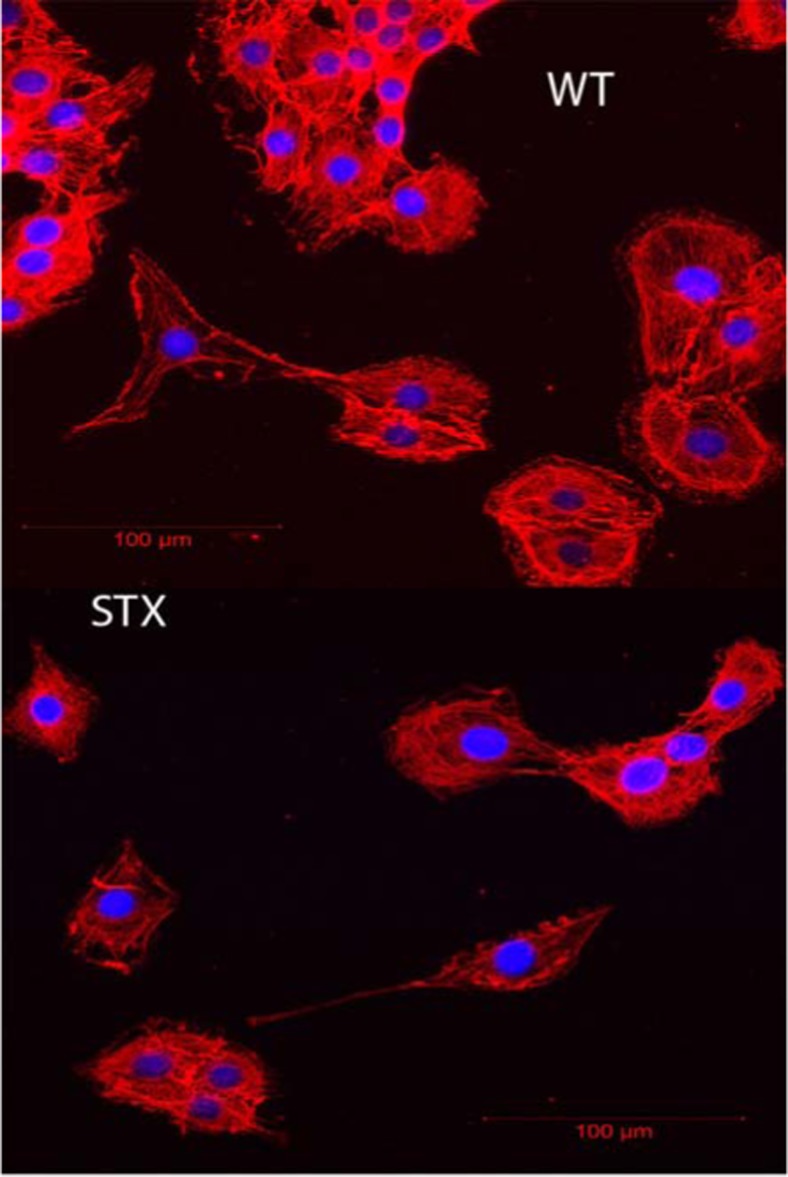
Laser confocal images of C6-WT and C6-STX cells immunolabelled for β-actin showing no discernible differences in labelling patterns

In this brief study, we have confirmed polySia expression on C6-STX cells and its absence on wild-type C6-WT cells. The AFM nano-mechanical experiments have demonstrated that the transfected glioma cells are mechanically distinct from the non-transfected wild type: a reduction in modulus and adhesion and increase in dissipative behaviour. This change in the mechanical behaviour of these isogenic cells is most likely to be due to the polySia expression on the cell membrane. Laser confocal microscopy images provided no discernible difference in actin within the cell types. However, the fact these cells were tested in a static condition may account for lack of differences in actin patterns, and future work will use the powerful model systems developed here to investigate the biomechanics in migrating cells. Furthermore, these results will provide additional mechanistic insights into the phenotypic changes associated with polySia expression, with important therapeutic implications.
